# Efficacy of Baduanjin for obesity and overweight: a systematic review and meta-analysis

**DOI:** 10.3389/fendo.2024.1338094

**Published:** 2024-06-11

**Authors:** Hainan Gao, Xue Li, Hongnan Wei, Xinxin Shao, Zili Tan, Shaowei Lv, Lijie Pan, Ting Yu, Qiuyan Ye, Haibo Zhang, Xiangyu Zhu

**Affiliations:** ^1^ School of Acupuncture-Moxibustion and Tuina, Beijing University of Chinese Medicine, Beijing, China; ^2^ Heilongjiang University of Traditional Chinese Medicine, Harbin, Heilongjiang, China

**Keywords:** obesity, overweight, Baduanjin, systematic review, meta-analysis

## Abstract

**Background:**

According to data from the World Health Organization (WHO), there is a significant public health issue regarding the increasing number of individuals affected by obesity and overweight on an annual basis. Therefore, it is imperative to urgently identify interventions that can effectively control and improve this condition. Baduanjin, as a medium-intensity exercise, appears a suitable approach for weight reduction among individuals with obesity. This paper aimed to provide a systematic review and meta-analysis of the efficacy of Baduanjin in addressing obesity and overweight, with the ultimate goal of assisting individuals with obesity in finding an effective, safe, and engaging method for weight reduction.

**Methods:**

We conducted a comprehensive search of multiple databases including PubMed, Cochrane Library, Web of Science, Embase, The China National Knowledge Infrastructure (CNKI), The Chinese Scientific Journal Database (VIP), The Chinese Biomedical Literature Database (CBM), and WanFang Database to identify relevant articles published from the inception of each database until September 2023. Specifically, we focused on randomized controlled trials (RCTs) investigating the effects of Baduanjin on weight reduction. Data from these studies were extracted and analyzed using appropriate statistical methods. In cases where there was no significant heterogeneity (*I* ^2^ < 50%, *p* > 0.1), we employed a fixed effects model for data synthesis; otherwise, a random effects model was selected. Funnel plots were used to assess publication bias, and the mean difference (MD) was reported as an indicator of treatment group differences.

**Results:**

A total of 420 participants were included in 10 studies. The MD results of the experimental group when compared with the control group were −3.69 (95%CI = −4.97 to −2.40, *p* < 0.001) for body weight (BW), −5.42 (95%CI = −6.56 to −4.28, *p* < 0.001) for body mass index (BMI), −1.36 (95%CI = −1.76 to −0.96, *p* < 0.001) for waist circumference (WC), −3.40 (95%CI = −4.43 to −2.37, *p* < 0.001) for hip circumference (HC), and −0.03 (95%CI = −0.04 to −0.02, *p* > 0.1) for the waist-to-hip ratio (WHR). All of the values in the experimental group showed significant difference. The results of the Egger’s test (*t* = 1.43, *p* = 0.190) suggest that there was no substantial bias present within the data analysis process. The safety profile revealed no adverse events reported across all 10 studies.

**Conclusion:**

Baduanjin could be effective in reducing weight, and the practice of Baduanjin has the potential to regulate BW, BMI, WC, HC, and WHR. However, further well-designed RCTs are still necessary to provide more robust evidence in the future.

**Systematic review registration:**

http://www.crd.york.ac.uk/PROSPERO/, identifier CRD42024513789.

## 1 Introduction

Obesity, which is characterized by the excessive accumulation or abnormal distribution of body fat (BF), currently poses a significant threat to public health (1). Recent data have revealed that approximately 34% of the global population is affected by overweight conditions, which warrants concern from all stakeholders (2, 3). Furthermore, the prevalence of obesity and overweight is associated with a multitude of diseases, including type 2 diabetes mellitus (T2DM), stroke, hypertension, dyslipidemia, osteoarthritis, and sleep apnea syndrome (4–9), as well as various types of cancer, such as cancers of the breast, liver, gallbladder, and colon (10–13). Therefore, there is an urgent need to explore effective weight loss strategies.

Studies have demonstrated physical exercise as a highly efficacious approach to weight management (14–17), and low-/medium-intensity exercise therapy has been identified as a suitable intervention for patients with obesity and overweight (18). However, the majority of low-/medium-intensity exercises tend to be monotonous and unstimulating. Consequently, individuals with obesity often struggle to maintain long-term adherence, thereby hindering their ability to achieve the desired weight loss outcomes. Hence, it is imperative to identify a safe, effective, and engaging exercise modality for individuals with obesity and overweight.

The Baduanjin exercise is a traditional Chinese qigong therapy that involves low-intensity, long-term aerobic exercises. It has been recognized as an effective approach to managing obesity and overweight for thousands of years (19). Comprising only eight distinct physical and mental exercises, Baduanjin is easier to learn compared with Tai Chi and is more suitable for individuals with obesity. This practice emphasizes the integration of body and mind through slow movements, focused concentration, and deep breathing accompanied by muscle stretching. Furthermore, it exhibits profound therapeutic effects on patients with various medical conditions. Currently, its popularity extends not only throughout Asia but also globally. Fang et al. (20), in their review, included several studies that demonstrated the safety of practicing Baduanjin, with minimal adverse reactions.

In the existing literature, numerous meta-analyses have primarily examined the efficacy and safety of Baduanjin intervention in T2DM, stroke, hypertension, dyslipidemia, osteoarthritis, and sleep apnea syndrome, as well as in certain types of cancer, such as breast cancer (21–27). However, the association between these diseases and obesity has been overlooked. If Baduanjin can be demonstrated as effective in reducing weight, then a Baduanjin intervention could help prevent the rapid deterioration of such conditions and offer enhanced protection for public health.

Currently, there is ongoing debate regarding the efficacy of clinical studies on the experimental use of Baduanjin for weight reduction. Furthermore, there is a scarcity of meta-analyses investigating the effects of Baduanjin in reducing weight. Yang et al. (28), in a published study in Frontiers in Endocrinology, conducted a systematic review on the effectiveness of traditional Chinese exercises for obesity. However, they encountered limitations due to the low quality of the studies included. Although their study categorized Baduanjin as a form of traditional Chinese exercise for systematic review purposes, it was still unable to definitively confirm its effectiveness in reducing weight.

Therefore, we conducted an extensive meta-analysis of research on Baduanjin interventions for obesity and overweight, including all available randomized controlled trials (RCTs) from diverse databases in various languages and exploring relevant subgroups. Our objective was to evaluate the efficacy of Baduanjin in the treatment of obesity and overweight.

## 2 Materials and methods

### 2.1 Sources of data

We conducted a comprehensive search of the following databases: PubMed, Web of Science, Cochrane Library, Embase, China National Knowledge Infrastructure (CNKI), Chinese Scientific Journals Database (VIP), Chinese Biomedical Literature Database (CBM), and Wanfang Database. The search included articles published from the inception of each database until September 1, 2023. In addition, we searched for grey literature in Open Grey, ClinicalTrials.gov, and WHO Clinical Trial Registration Center. In cases where duplicate publications were identified, the most recent and complete trials were selected.

The search strategy for PubMed is presented in **Table 1**.

**Table 1:** Search strategy used in PubMed.

number	Search terms
#1	"Obesity"[Mesh] Sort by; Most Recent
#2	Obese[Title/Abstract]
#3	("Obesity"[Mesh]) OR (obese[Title/Abstract])
#4	baduanjin[Title/Abstract]
#5	Randomized controlled trials[MeSH Terms]
#6	(((((randomly) OR (randomized) ) OR (RCT)) OR (Randomized clinical trials)) OR (trials)) OR (Random allocation)
#7	#5 AND #6
#8	#3 AND #4 AND #7

### 2.2 Inclusion and exclusion criteria

Our meta-analysis adhered to the guidelines outlined in the Preferred Reporting Items for Systematic Reviews and Meta-Analyses (PRISMA) Statement and has been duly registered at the International Prospective Register of Systematic Reviews under registration number CRD42024513789 (www.crd.york.ac.uk/PROSPERO/).

The inclusion criteria were as follows: 1) the study must be an RCT; 2) age of participants >18 years; 3) the diagnostic criteria for obesity/overweight used in the study were based on region and ethnicity (25.0 kg/m^2^ for Asia Pacific and 30.0 kg/m^2^ for adults in the Americas); and 4) the experimental group was required to include Baduanjin exercises. The control group had no experimental methods other than Baduanjin.

With regard to age selection, according to the General Administration of Sports of China, the respiratory control ability of minors is not fully developed, which hinders their comprehension of the intricate breathing techniques and mental skills involved in Baduanjin. Moreover, it is inconsistent with the criteria for daily adult interaction. Therefore, the eligibility for study participation was restricted to individuals aged 18 years or older.

The exclusion criteria were as follows: 1) did not meet the BMI of obesity according to the region and ethnic group; 2) aged <18 years; 3) pregnant women; and 4) the experimental group did not do Baduanjin or the control group also did Baduanjin.

### 2.3 Quality assessment

The quality of the included studies was assessed using the revised Cochrane Risk of Bias tool (ROB 2.0) outlined in the Cochrane Handbook for Systematic Reviews of Interventions. The following parameters were evaluated: the randomization process, deviations from the intended experimental protocols, missing outcome data, the measurement accuracy of the outcomes, and the selection criteria for the reported results. Each item was categorized as having a high risk of bias, some concerns (unclear risk of bias), or low risk of bias. The overall assessment considering all of these factors resulted in the evaluation of overall bias.

### 2.4 Statistical analysis

STATA software was used to conduct the meta-analysis of the selected studies. The mean difference (MD) was utilized as a continuous effect size indicator for the variables, employing 95% confidence intervals (95%CI) in forest plots. Sensitivity analysis and subgroup analysis were performed to investigate the sources of heterogeneity. For the sensitivity analysis, the one-by-one elimination method was used to identify the source of heterogeneity by re-estimating the combined effect. For the subgroup analysis, studies were categorized into different subgroups based on duration and whether these subgroup factors can be proven as the source of heterogeneity.

### 2.5 Study selection and data extraction

Two independent investigators (HNG and XXS) read the title, abstract, and full text; screened the literature according to the inclusion and exclusion criteria; and cross-checked the results. Data were extracted separately by a third investigator (QYY). In the case of a disagreement, a fourth investigator (HBZ) was consulted. The data extracted from the included studies were as follows: first author, date of publication, the mean age or age range of the study participants, the study sample size, the interventions used, the intervention and control groups included, the duration of the study, adverse events, and outcomes.

## 3 Results

### 3.1 Retrieved results

**Figure 1:**
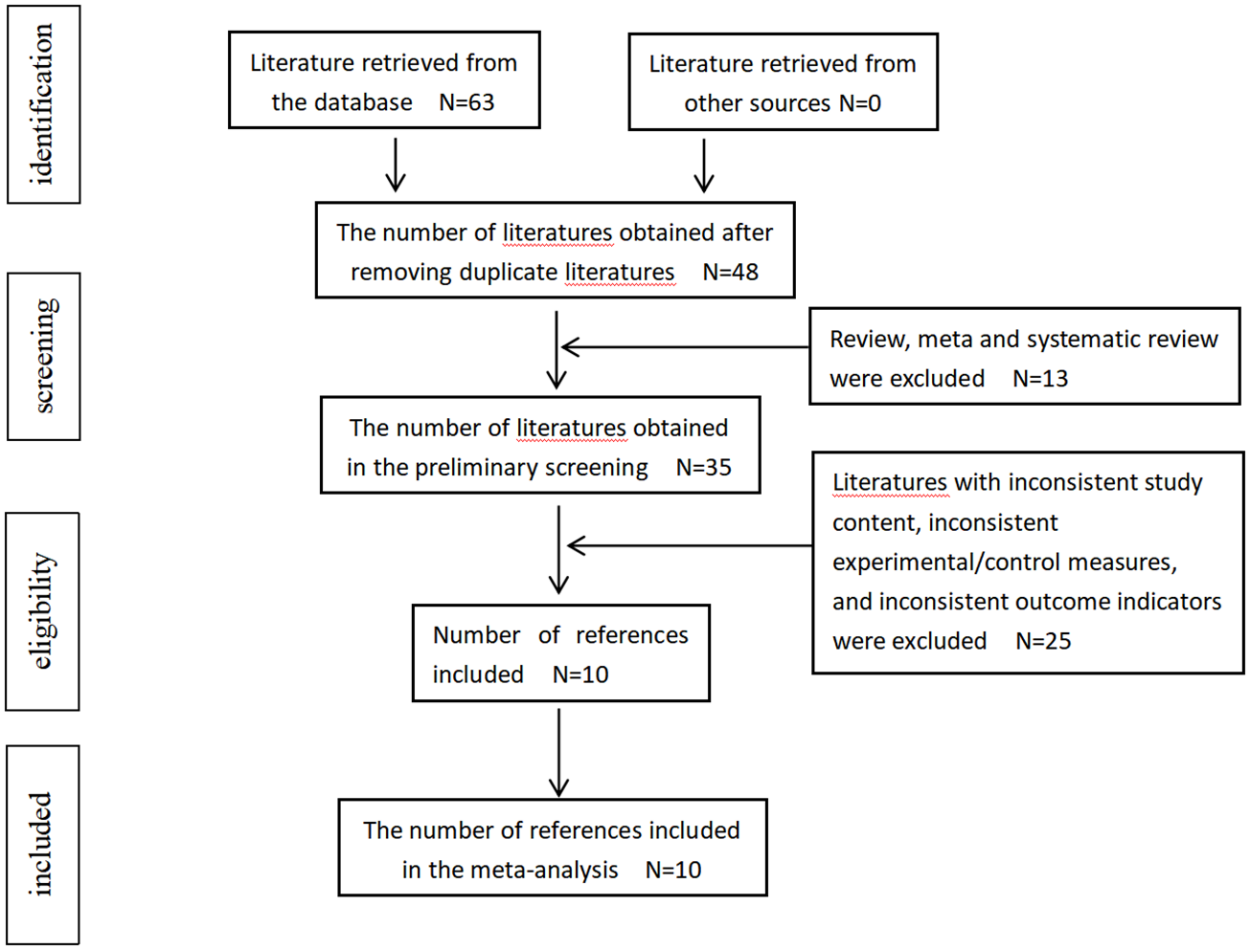
Flowchart of the search results and the selection details.

The initial search yielded a total of 63 articles, from which 15 were excluded due to duplication. After carefully reviewing the titles and abstracts, an additional 13 articles were eliminated. A comprehensive evaluation was conducted on a total of 35 articles, among which 10 were included in this meta-analysis (29–38). There were no additional studies identified through manual search. The study selection process is illustrated in **Figure 1**.

### 3.2 Research characteristics

The meta-analysis included 10 studies, with a total of 420 participants. The outcomes studied were as follows: 1) body weight (BW); 2) body mass index (BMI); 3) waist circumference (WC); 4) hip circumference (HC); and 5) waist-to-hip ratio (WHR). The main characteristics of the 10 articles are summarized in **Table 2**.

**Table 2:** Summary of the main characteristics.

Number	Author, year	Age (years)	Sample size (experimental/control)	Experimental group	Control group	Duration (weeks)	Adverse reactions	Outcomes
1	Yan Jiang, 2020 (30)	64.18 ± 4.09	23 (12/11)	5 × 45 min/week	Special lecture 1 × 60 min/week	12	Not mentioned	BW, BMI
2	Tao Liu, 2018 (35)	57.2 ± 5.4	40 (20/20)	6 × 90 min/week	Daily activities	24	Not mentioned	BW, BMI, WC, HC, WHR
3	Yan Rao, 2020 (34)	18–21	32 (16/16)	4 × 90 min/week	Daily activities	16	Not mentioned	BW
4	Dawei Zhang, 2023 (33)	19.46 ± 0.68	68 (34/34)	7 × 45 min/week	Daily activities	8	Not mentioned	BW, BMI, WC, HC
5	Xiaoqiang Zhang, 2008 (38)	41 ± 5.61	23 (13/10)	5 × 50 min/week	Daily activities	10	Not mentioned	BW, WC
6	Zhongsun Yu, 2017 (36)	18–23	46 (23/23)	5 × 60 min/week	Daily activities	16	Not mentioned	BW, BMI, WC, HC, WHR
7	Dongming Jia, 2022 (31)	36.4 ± 3.55	36 (18/18)	5 × 60 min/week	Daily activities	8	No adverse reaction	BW, BMI, WC, HC
8	Ning Wang, 2019 (37)	22.79 ± 1.719	68 (34/34)	Baduanjin 7 × 2 × 15 min/week + acupoint embedding	Acupoint embedding	8	No adverse reaction	BW, BMI, WC, HC, WHR
9	Biqi Pan, 2013 (29)	37.21 ± 8.81	64 (32/32)	Baduanjin 7 × 2 × 30 min/week + acupoint embedding	Acupoint embedding	12	Not mentioned	BW, BMI, WC, HC, WHR
10	Yong Zhou, 2013 (32)	Null	20 (10/10)	7 × 2 × 45 min/week	Grape sugar solution 7 × 2 × 45 ml/kg per week	14	Not mentioned	BW, BMI

BW, body weight; BMI, body mass index; WC, waist circumference; HC, hip circumference; WHR, waist-to-hip ratio.

### 3.3 Quality of evidence

**Figure 2:**
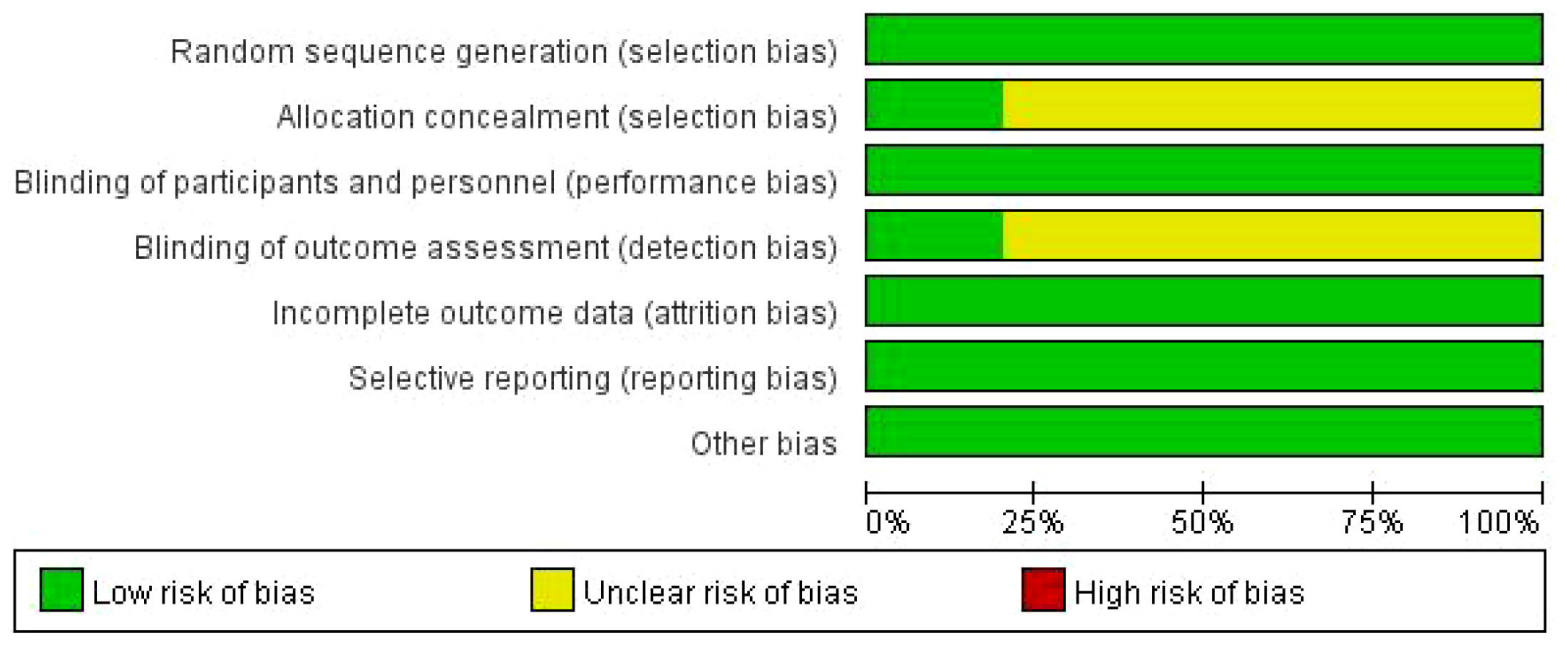
Risk of bias graph (Baduanjin) on body weight (BW).

The results of the bias risk assessment for all included studies are summarized in the figure below. With regard to bias in the randomization process, all 10 studies mentioned employing random assignment, with two of the studies specifically mentioning the use of numerical random table assignment. The baseline characteristics of the subjects in both the experimental and control groups were consistent in terms of demographic data and clinical characteristics. In terms of bias arising from deviations from intended experiments, missing outcome data, and measurement of outcomes, all 10 RCTs were considered to have a low risk. Due to the unique nature of Baduanjin training, achievement of blinding for both participants and staff was challenging; therefore, lack of blinding was not considered as a source of bias. For hidden bias related to assignment concealment, eight studies were deemed to have an ambiguous risk, while two studies were classified as having a low risk. The risk of bias graph (Baduanjin) on BW is shown in **Figure 2**.

The factors contributing to bias included: 1) inadequate reporting of randomization; lack of specific description or incorrect method used; 2) absence or unclear reporting of allocation concealment for the randomization protocol; 3) insufficient clarity in reporting the implementation of blinding; and 4) incomplete reporting on the study protocol registration or unclear discussion of the reported entries.

### 3.4 Search results

#### 3.4.1 Body weight

**Figure 3:**
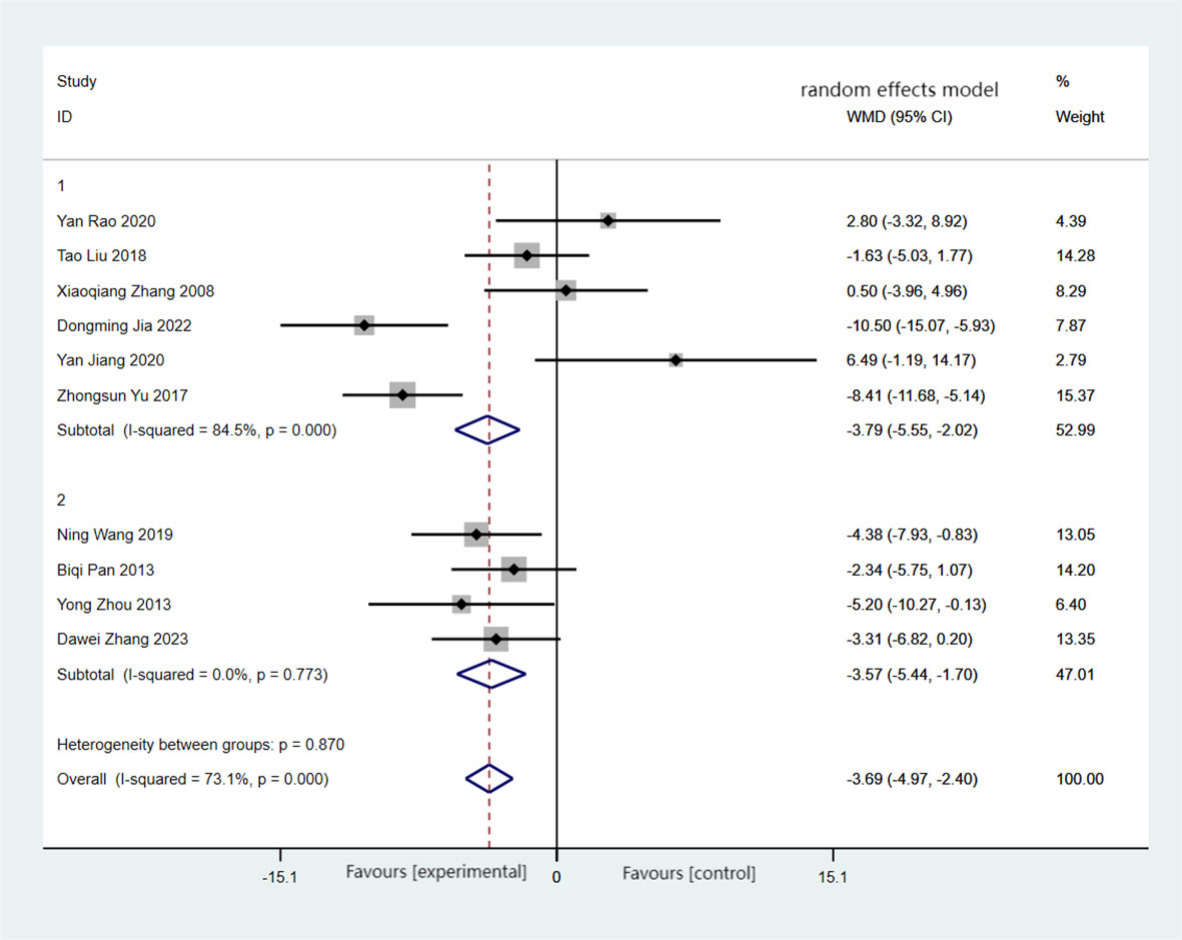
Forest plots and subgroup analysis of the training time for Baduanjin on body weight (BW). Subgroup 1, non-daily training; subgroup 2, daily training.

There were 10 articles reporting on BW, encompassing a total of 420 cases, with the experimental group comprising 212 cases and the control group comprising 208 cases. The comprehensive findings revealed that, in comparison to the control group, the experimental group exhibited significant weight loss (MD = −3.69, 95%CI = −4.97 to −2.40), albeit with higher heterogeneity (*I* ^2^ = 84.5%, *p* < 0.001). Forest plots and subgroup analysis of the training time for Baduanjin on body weight (BW) in **Figure 3**.

Subgroup analysis revealed that the heterogeneity between the experimental group and the control group disappeared when excluding the non-daily training group (MD = −3.57, 95%CI = −5.44 to −1.70, *I* ^2^ = 0, *p* = 0.773). Regardless of whether the training time was daily or non-daily, there were significant differences in BW between the two groups (39).

The results of the meta-analysis conducted using the random effects model demonstrated a statistically significant reduction in BW in the experimental group compared with the control group, with a MD of 3.69. These findings suggest that Baduanjin could be an effective intervention for reducing weight.

#### 3.4.2 Body mass index

**Figure 4:**
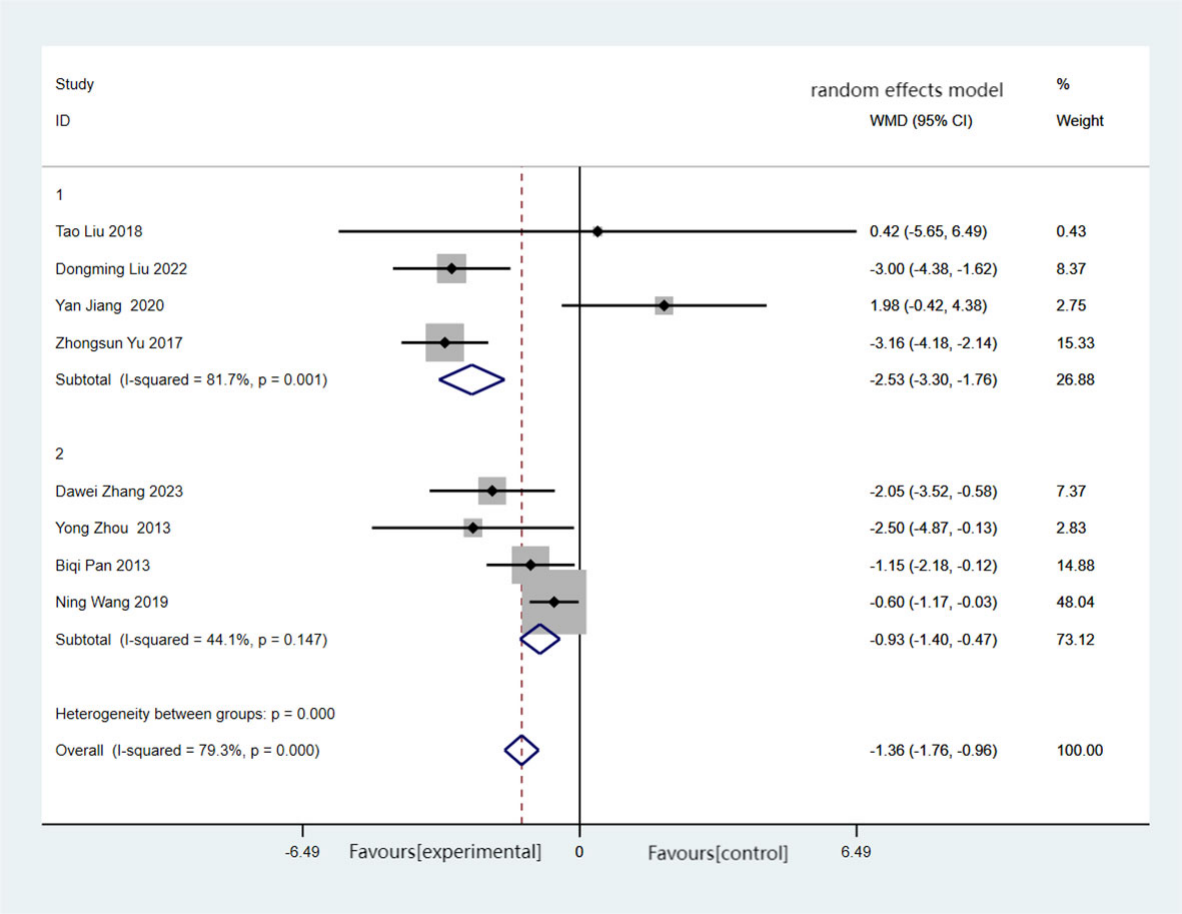
Forest plots and subgroup analysis of the training time for Baduanjin on body mass index (BMI). Subgroup 1, non-daily training; subgroup 2, daily training.

BMI was reported in eight of the included studies, encompassing a total of 365 cases, with 183 cases in the experimental group and 182 cases in the control group. The combined findings revealed that, compared with the control group, the experimental group demonstrated a significant reduction in BMI (MD = −1.36, 95%CI = −1.76 to −0.96), albeit with substantial heterogeneity (*I* ^2^ = 79.3%, *p* < 0.001). Forest plots and subgroup analysis of the training time for Baduanjin on body mass index (BMI) in **Figure 4**.

Subgroup analysis revealed that the heterogeneity between the experimental group and the control group disappeared when excluding the non-daily training group (MD = −0.93, 95%CI = −1.40 to −0.47, *I* ^2^ = 44.1%, *p* = 0.147). Regardless of daily or non-daily training, there were significant differences in BMI between the two groups.

The results of the meta-analysis using the random effects model revealed a statistically significant reduction in BMI for the experimental group compared with the control group, with a MD of 1.36. This suggests that Baduanjin training could be an effective intervention for reducing BMI.

#### 3.4.3 Waist circumference

**Figure 5:**
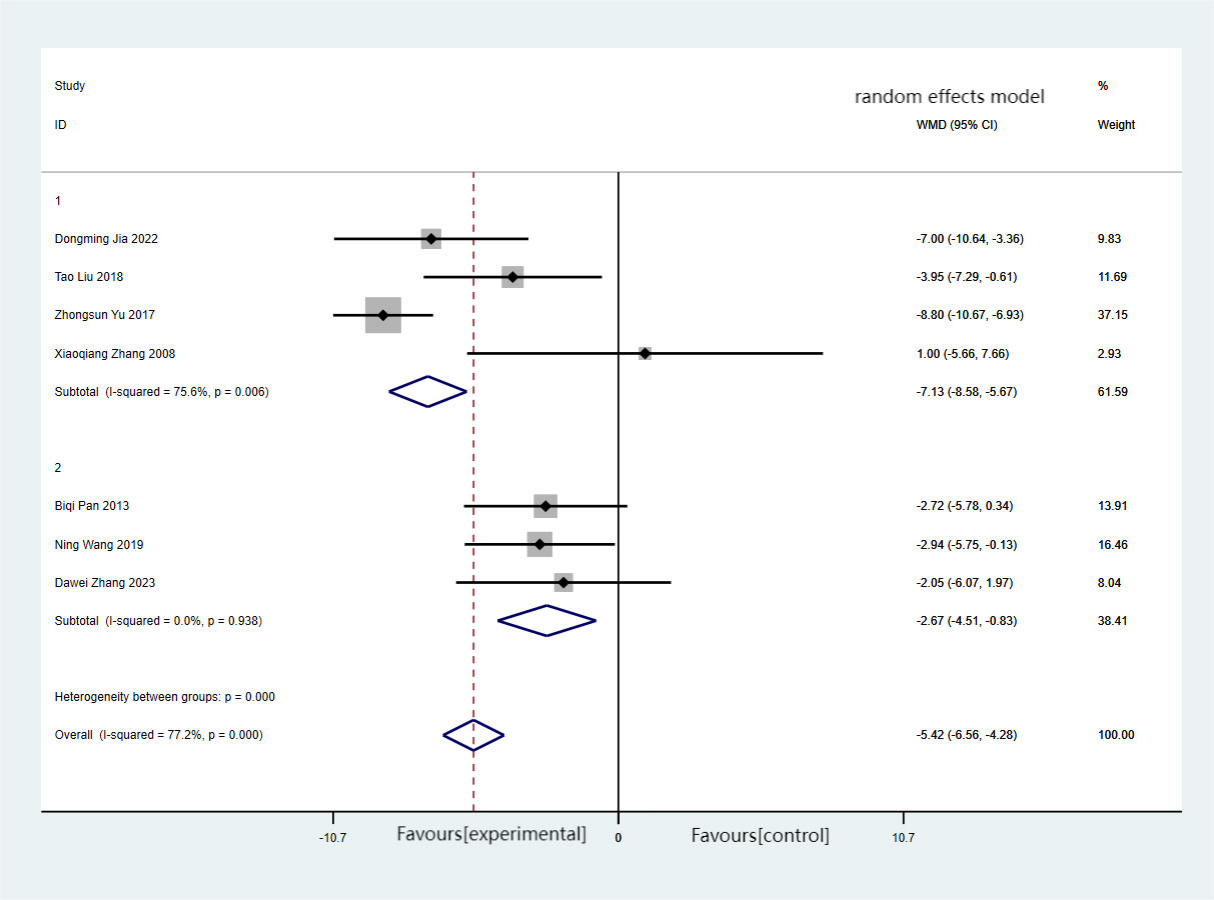
Forest plots and subgroup analysis of the training time for Baduanjin on waist circumference (WC). Subgroup 1, non-daily training; subgroup 2, daily training.

There were a total of seven articles reporting on WC, encompassing 345 cases in total. Among these, the experimental group consisted of 174 cases, while the control group had 171 cases. Comprehensive findings indicated that the experimental group effectively reduced WC (MD = −5.42, 95%CI = −6.56 to −4.28), with significant heterogeneity (*I* ^2^ = 77.2%, *p* < 0.001). Forest plots and subgroup analysis of the training time for Baduanjin on waist circumference (WC) in **Figure 5**.

Subgroup analysis revealed that the heterogeneity between the experimental and control groups vanished when excluding the non-daily training group (MD = −2.67, 95%CI = −4.51 to −0.83, *I* ^2^ = 0, *p* = 0.938). Notably, significant differences in WC were observed between the two groups irrespective of whether the training was conducted daily or non-daily.

The results of the meta-analysis conducted using the random effects model demonstrated a statistically significant reduction in WC of 5.42 in the experimental group compared with the control group. These findings suggest that Baduanjin training could effectively contribute to reducing WC.

#### 3.4.4 Hip circumference

**Figure 6:**
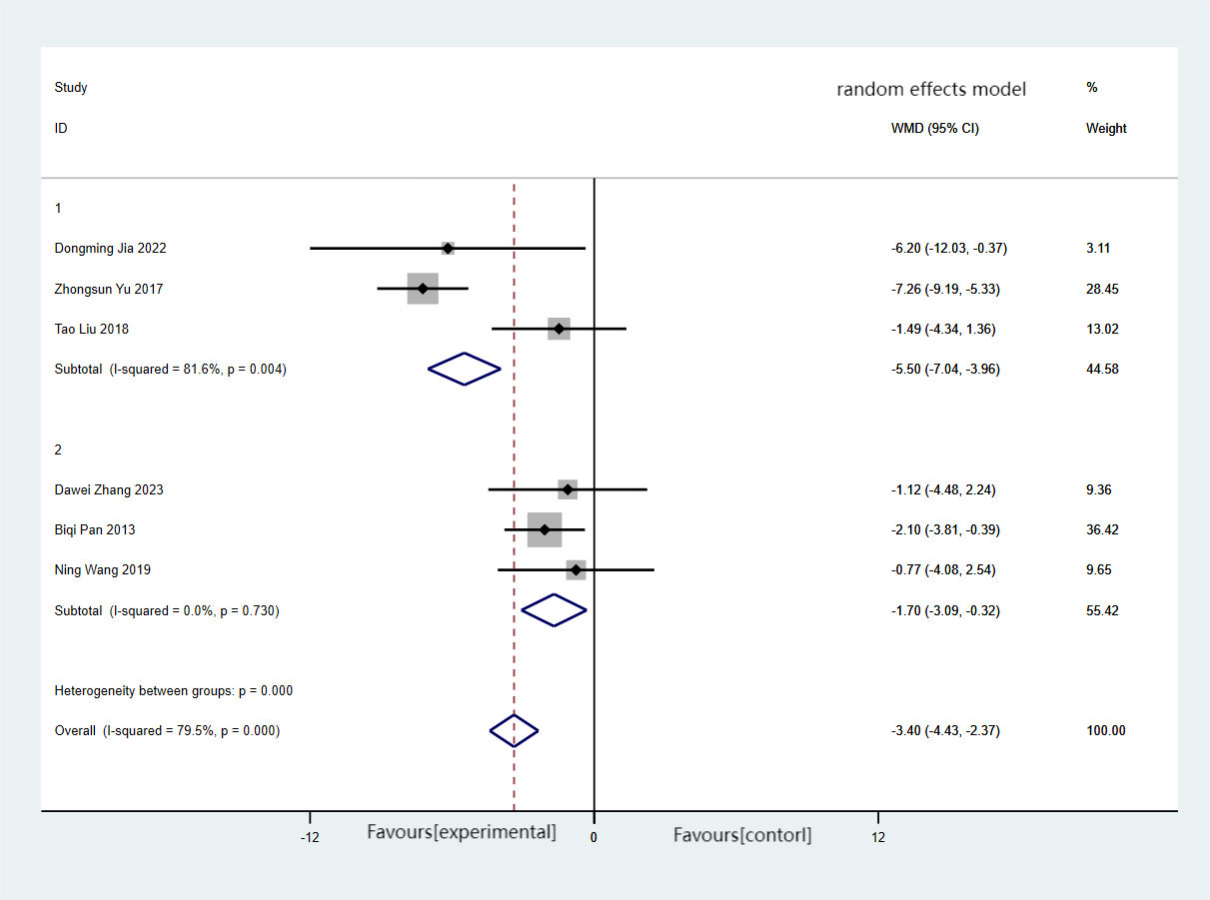
Forest plots and subgroup analysis of the training time for Baduanjin on hip circumference (HC). Subgroup 1, non-daily training; subgroup 2, daily training.

There were six articles reporting on HC, encompassing a total of 322 cases, with 161 in the treatment group and 161 in the control group. The combined findings demonstrated a significant reduction in HC in the treatment group compared with the control group (MD = −3.40, 95%CI = −4.43 to −2.37), albeit with substantial heterogeneity (*I* ^2^ = 79.5%, *p* < 0.001). Forest plots and subgroup analysis of the training time for Baduanjin on hip circumference (HC) in **Figure 6**.

The subgroup analysis revealed that the heterogeneity between the experimental and control groups disappeared when excluding the non-daily training group (MD = −1.70, 95%CI = −3.09 to −0.32, *I* ^2^ = 0, *p* = 0.730). Regardless of whether the training time was daily or non-daily, there were significant differences in HC between the two groups.

The results of the meta-analysis conducted using the random effects model demonstrated a statistically significant reduction in HC (MD = −3.40) for the experimental group compared with the control group. These findings suggest that Baduanjin training could be an effective intervention for reducing HC.

#### 3.4.5 Waist-to-hip ratio

**Figure 7:**
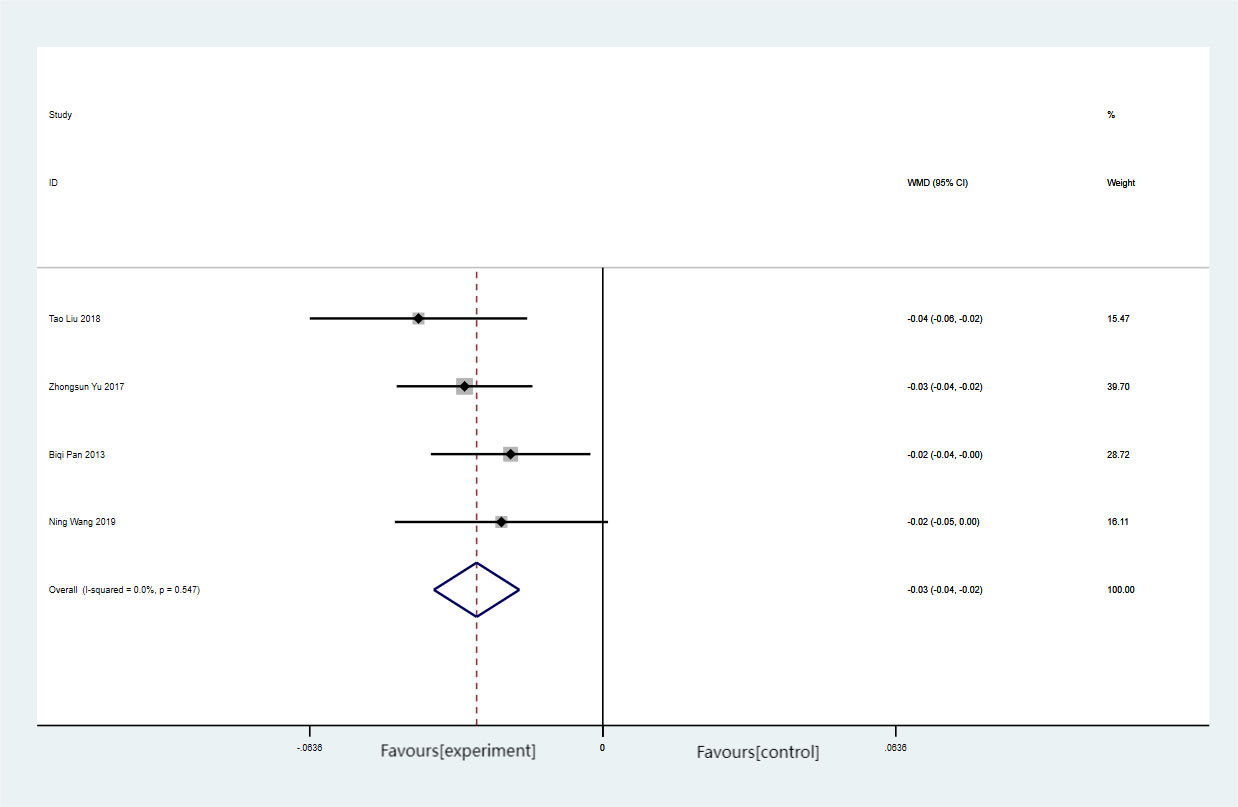
Forest plots of the training time for Baduanjin on the waist-to-hip ratio (WHR).

There were a total of six articles reporting on the WHR, encompassing 218 cases, with 109 cases in both the experimental and control groups. No heterogeneity was observed among the included studies (*p* = 0.547, *I* ^2^ = 0%), leading to the adoption of a fixed effects model. The findings revealed a MD of 0.03 (95% CI = −0.04 to −0.02). Forest plots of the training time for Baduanjin on the waist-to-hip ratio (WHR) in **Figure 7**.

Meta-analysis of the fixed effects model revealed a statistically significant reduction in the WHR of 0.03 in the experimental group compared with the control group (*p* > 0.1). This suggests that the experimental intervention had a superior impact on reducing the WHR when compared with the control group.

### 3.5 Evaluation of publication bias

**Figure 8:**
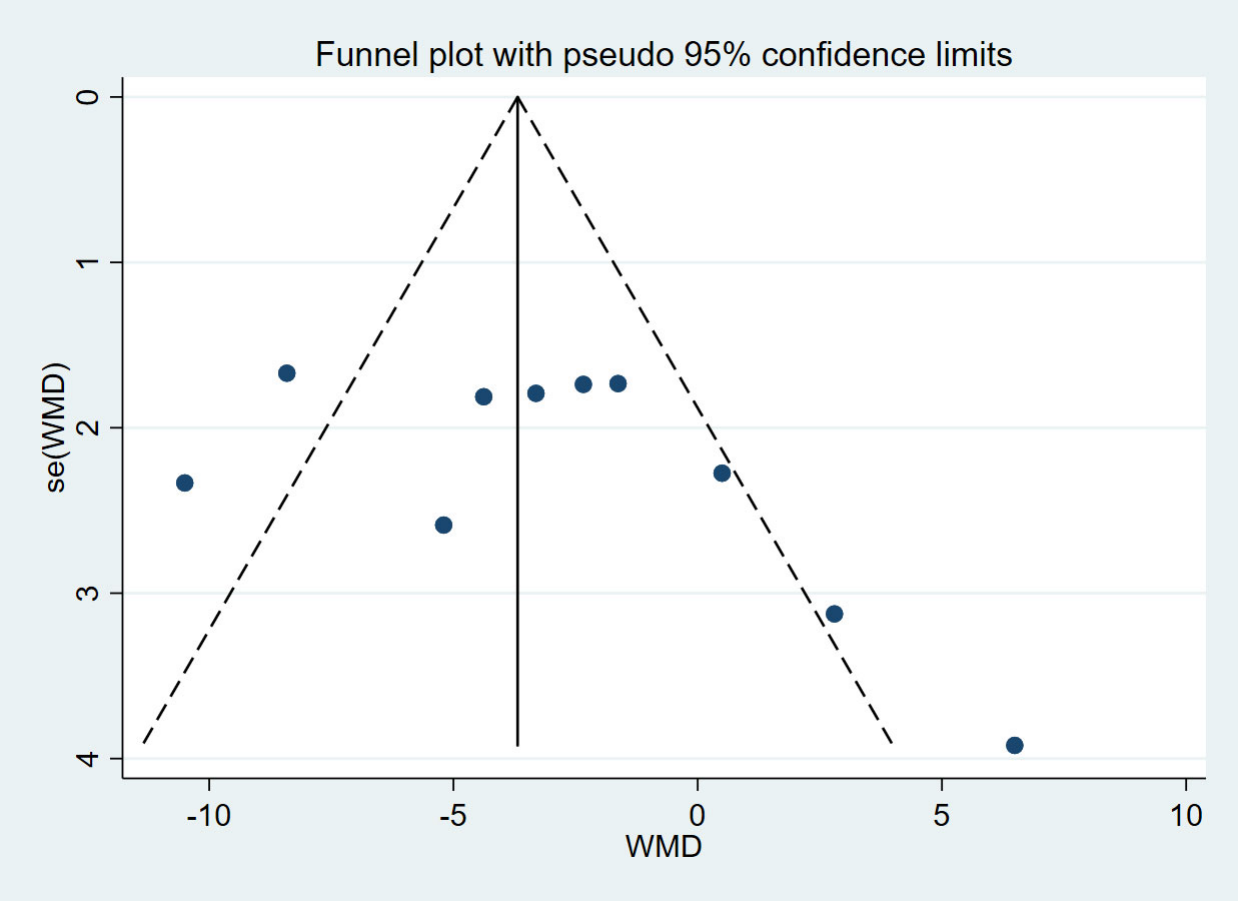
Funnel plot for body weight (BW) included in the meta-analysis.

The presence of publication bias in BW was assessed using funnel plots based on the 10 studies. It was evident that the funnel plot exhibited symmetrical distribution, indicating no significant publication bias. Funnel plot for body weight (BW) in **Figure 8**. The analysis results revealed that the Egger’s test for BW (*t* = 1.43, *p* = 0.190) demonstrated no substantial evidence of publication bias, thereby exerting no influence on the findings.

### 3.6 Grading of the quality of evidence

The quality of evidence was assessed using the GRADE (Grades of Recommendation, Assessment, Development, and Evaluation) method and was categorized into high, moderate, low, or very low grade. The assessment criteria employed in the GRADE method encompassed risk of bias, inconsistency, indirectness, imprecision, and publication bias. A specific grade was assigned to each outcome. The results for GRADE in this study are shown in **Table 3**.

**Table 3:** Summary of the results for GRADE (Grades of Recommendation, Assessment, Development, and Evaluation).

Certainty assessment							№ of patients		Effect	Certainty	Importance
№ of studies	Study design	Risk of bias	Inconsistency	Indirectness	Imprecision	Other considerations	Experimental group	Control group	Absolute(95% CI)		
BW	randomised trials	serious^a^	serious^b^	not serious	not serious	none	212	208	MD 3.69 lower (4.97 lower to 2.4 lower)	⨁⨁○○ Low	Important
BMI	randomised trials	serious^a^	serious^b^	not serious	not serious	none	183	182	MD 1.36 lower (1.76 lower to 0.96 lower)	⨁⨁○○ Low	Important
WC	randomised trials	serious^a^	serious^b^	not serious	not serious	none	174	171	MD 5.42 lower (6.56 lower to 4.28 lower)	⨁⨁○○ Low	Important
HC	randomised trials	serious^a^	serious^b^	not serious	not serious	none	161	161	MD 3.4 lower (4.43 lower to 2.37 lower)	⨁⨁○○ Low	Important
WHR	randomised trials	serious^a^	not serious	not serious	serious^c^	none	109	109	MD 0.03 lower (0.04 lower to 0.02 lower)	⨁⨁○○ Low	Important

BW, body weight; BMI, body mass index; WC, waist circumference; HC, hip circumference; WHR, waist-to-hip ratio; CI, confidence interval; MD, mean difference.

a Lacking blinding or unclear allocation.

b Substantial heterogeneity.

c Small sample size or only a few included articles.

⨁ represents the evidence quality rating, ⨁⨁ is low.

⨁⨁○○ means “low” certainty.

### 3.7 Sensitivity analysis

**Figure 9:**
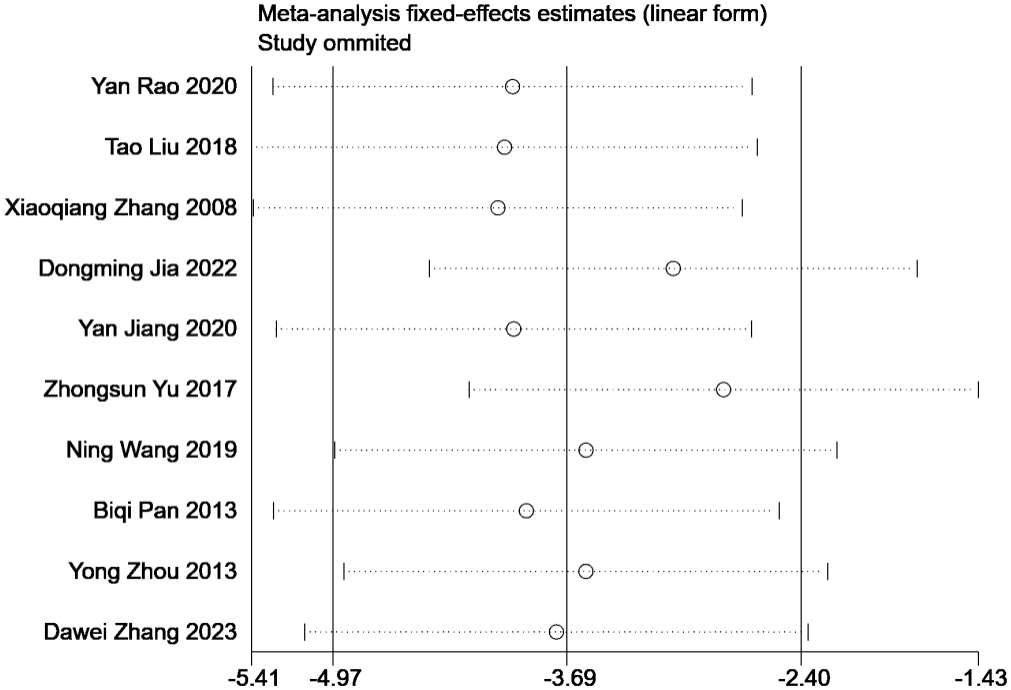
Sensitivity analysis of the outcome indicators.

The sensitivity of the outcome indicators was analyzed using the case-by-case exclusion method. The results demonstrated no significant changes, thereby indicating the robustness of the findings. Sensitivity analysis of the outcome indicators in **Figure 9**.

## 4 Discussion

The present study included 10 research studies for a meta-analysis examining the efficacy of Baduanjin training in obesity and overweight. These studies consisted of one high-quality article and nine medium-quality articles. The findings demonstrated that the experimental group undergoing Baduanjin training exhibited superior outcomes compared with the control group across various measures, which included BW, BMI, WC, HC, and WHR. This suggests that a Baduanjin intervention might hold promise in reducing weight.

The Baduanjin exercise is characterized by its low intensity, long duration, and uninterrupted rhythm. Its low-intensity and prolonged nature contribute to the utilization of BF. The training primarily targets the head, shoulders, back, waist, and limbs, effectively promoting blood circulation. In addition, through deep breathing techniques that enhance oxygen intake and metabolism acceleration, it aids in the consumption of BF. Research (40) indicates that regular participation in moderate aerobic exercise can effectively trigger mechanisms for fat oxidation energy while reducing adipocyte volume to achieve weight reduction effects. Liu et al. (41) further demonstrated that Baduanjin exercise improves the muscle glucose absorption capacity and effectively regulates the sugar intake and fat accumulation. Moreover, it also controls emotions and functional activities to regulate autonomic nervous function while influencing the adrenal medulla function and enhancing metabolism. Furthermore, the gentle movements of the limbs facilitate sugar breakdown and consumption.

Simultaneously, obesity is frequently correlated with diabetes, hypertension, hyperlipidemia, and other ailments (35). Existing research indicates that Baduanjin also diminishes the levels of fasting plasma glucose (FPG), hemoglobin A1C (HbA1C), triglycerides (TGs), total cholesterol (TC), low-density lipoprotein (LDL), high-density lipoprotein (HDL), human serum retinal-binding protein 4(RBP4), serum leptin, and adiponectin, among others (32).

Jiding et al. (42) also confirmed the positive impact of Baduanjin on BMI, further demonstrating its ability to elevate abdominal temperature, to stimulate specific acupuncture points, and to enhance metabolism and blood circulation. Yang et al. (25) conducted a meta-analysis of experiments on Chinese traditional exercise in obesity, where Baduanjin was found to be effective in reducing weight after experimentation. However, this analysis only included two articles on Baduanjin due to significant heterogeneity, potential bias risk, and low article quality. Therefore, in this study, we incorporated 10 RCTs of medium or higher quality that investigated the effects of Baduanjin intervention on weight reduction for a more compelling outcome.

The results of the subgroup analysis indicated that neither daily nor non-daily exercise was a significant source of heterogeneity in the studies included.

Of the 10 papers included in this study, two mentioned the experimental use of Baduanjin with no reported adverse events, while the remaining eight made no mention of any adverse events. Fang et al. (20) also investigated the safety profile of Baduanjin exercise in comparison to other exercises. Overall, the results indicated that Baduanjin exercise is a safer option for weight loss. However, due to the limited number of literature samples included, more comprehensive RCTs are still required to establish the safety of Baduanjin exercise for weight reduction in the future.

Our systematic evaluation has some limitations. Firstly, the sample size of this meta-analysis is relatively small, which could have introduced unknown bias due to incomplete data and potentially constrained our results. Secondly, there were significant variations in the experimental regimens with regard to the study duration (ranging from 8 weeks to 24 months), frequency (4–14 times per week), and the duration of each exercise session (from 15 to 60 min), among other factors. Thirdly, the control group received different interventions, which could have contributed to the heterogeneity observed in the studies. Despite these limitations, this meta-analysis provided valuable insights into the association between Baduanjin and weight reduction.

Building on the current study, future studies should increase the sample size of clinical observations in order to provide more objective clinical evidence. 1) More trials are needed to compare the effects of Baduanjin treatment and other treatments on obesity and overweight. 2) Shedding events during the treatment with Baduanjin should be reported in detail to prevent bias and to better assess the validity of the data.

Although the studies included in this paper had certain limitations, the exploration of the weight loss effects of the Eight Section Brocade can bring more widespread attention to the practice of Eight Section Brocade exercises on a global scale. In the future, there will be more scholars using larger-scale and more comprehensive RCTs to prove its effectiveness.

## 5 Conclusion

In conclusion, Baduanjin exercise has demonstrated effectiveness in reducing BW, BMI, WC, HC, and WHR among individuals with obesity and overweight. The statistically significant results indicated its efficacy in managing obesity and in facilitating weight loss in patients with obesity and overweight. Therefore, it is recommended to consider incorporating the Baduanjin exercise method in the treatment and prevention of obesity and overweight. Moreover, compared to Tai Chi and yoga, Baduanjin is more accessible to a wider range of individuals due to its ease of learning.

## Data availability statement

The datasets presented in this study can be found in online repositories. The names of the repository/repositories and accession number(s) can be found in the article/**Supplementary Material**.

## Author contributions

HG: Writing – original draft. XL: Writing – review & editing, Data curation. HW: Writing – original draft, Writing – review & editing, Data curation, Validation. XS: Writing – review & editing, Software. ZT: Writing – review & editing, Methodology. SL: Writing – review & editing, Formal analysis. LP: Writing – review & editing, Conceptualization. TY: Writing – review & editing, Validation. QY: Writing – review & editing, Project administration. HZ: Writing – review & editing. XZ: Writing – original draft, Funding acquisition.
