# Supplementary figures and images for: Efficacy of Baduanjin for obesity and overweight: a systematic review and meta-analysis
Source: Front Endocrinol (Lausanne). 2024 Jun 11;15:1338094. doi: 10.3389/fendo.2024.1338094 (PMC11196404; doi:10.3389/fendo.2024.1338094)

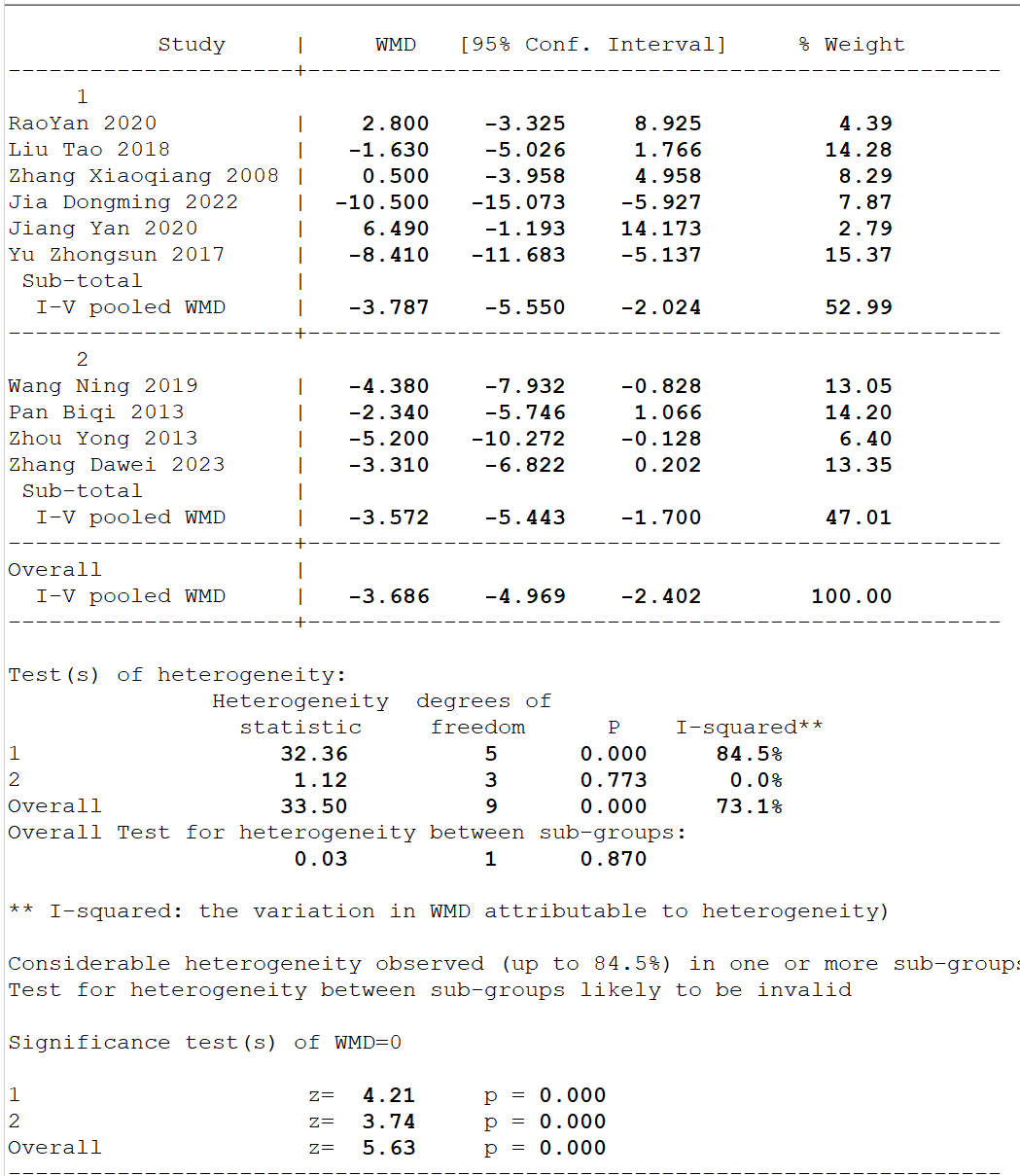

Supplement: Supplementary file 1 [file Image_1.jpeg]

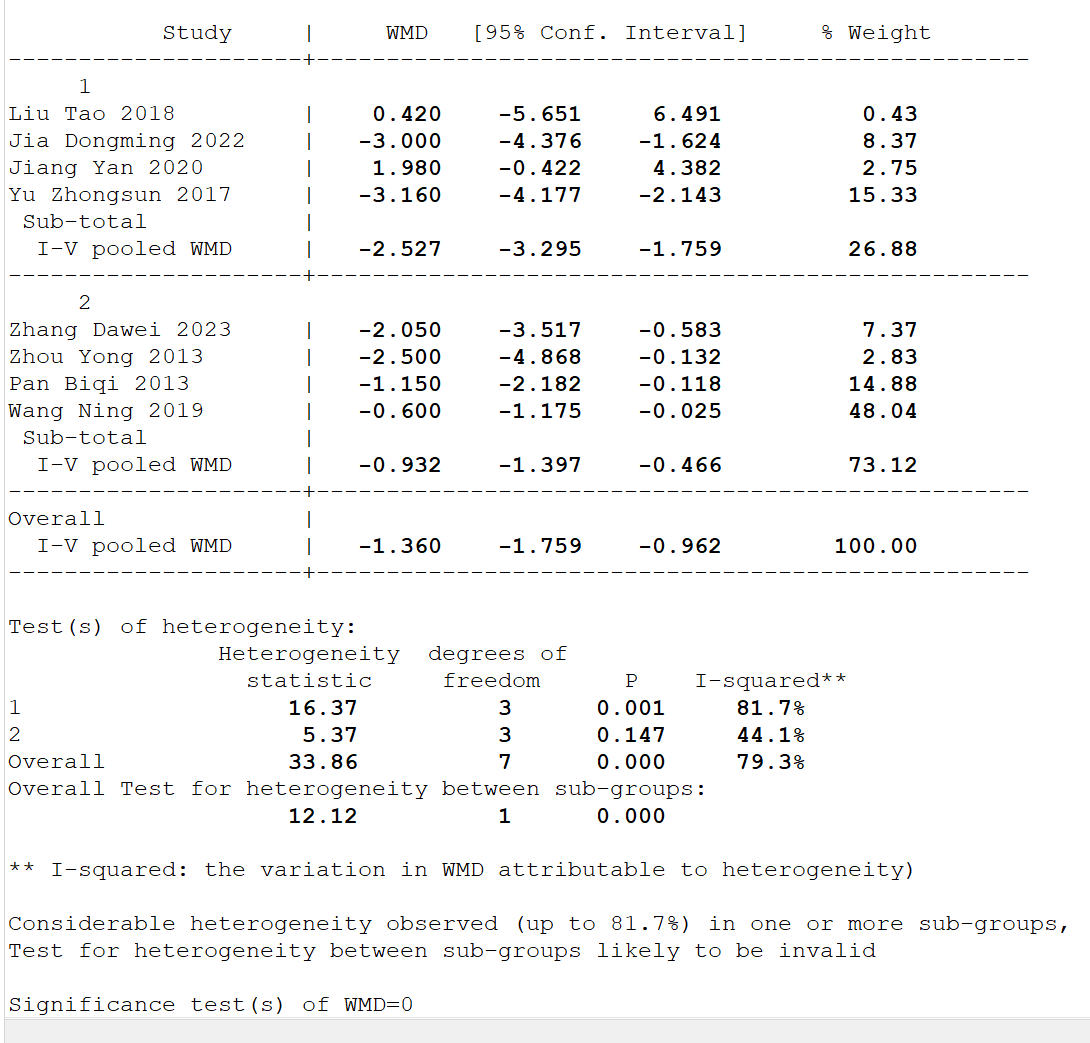

Supplement: Supplementary file 2 [file Image_2.jpeg]

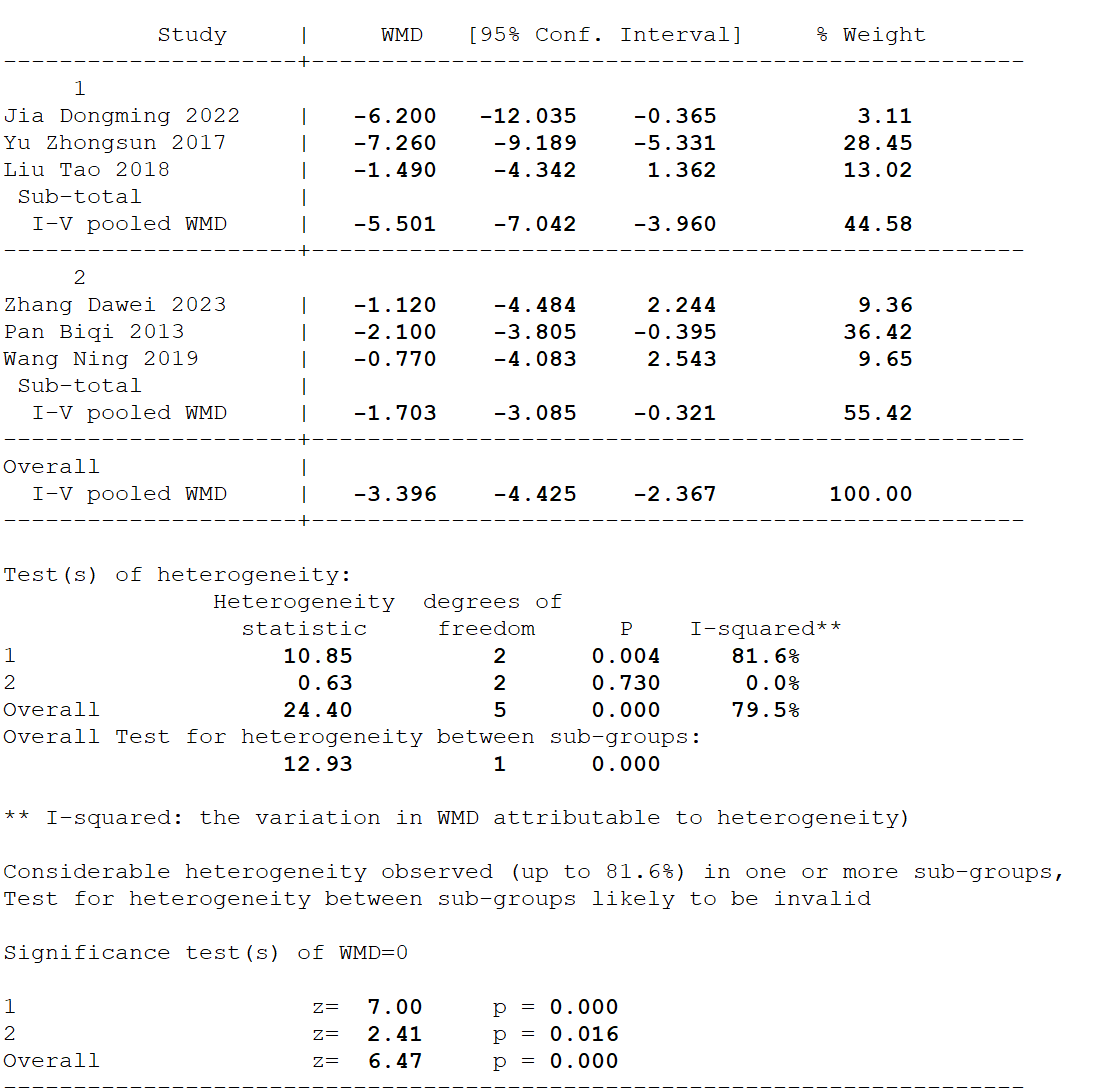

Supplement: Supplementary file 3 [file Image_3.jpeg]

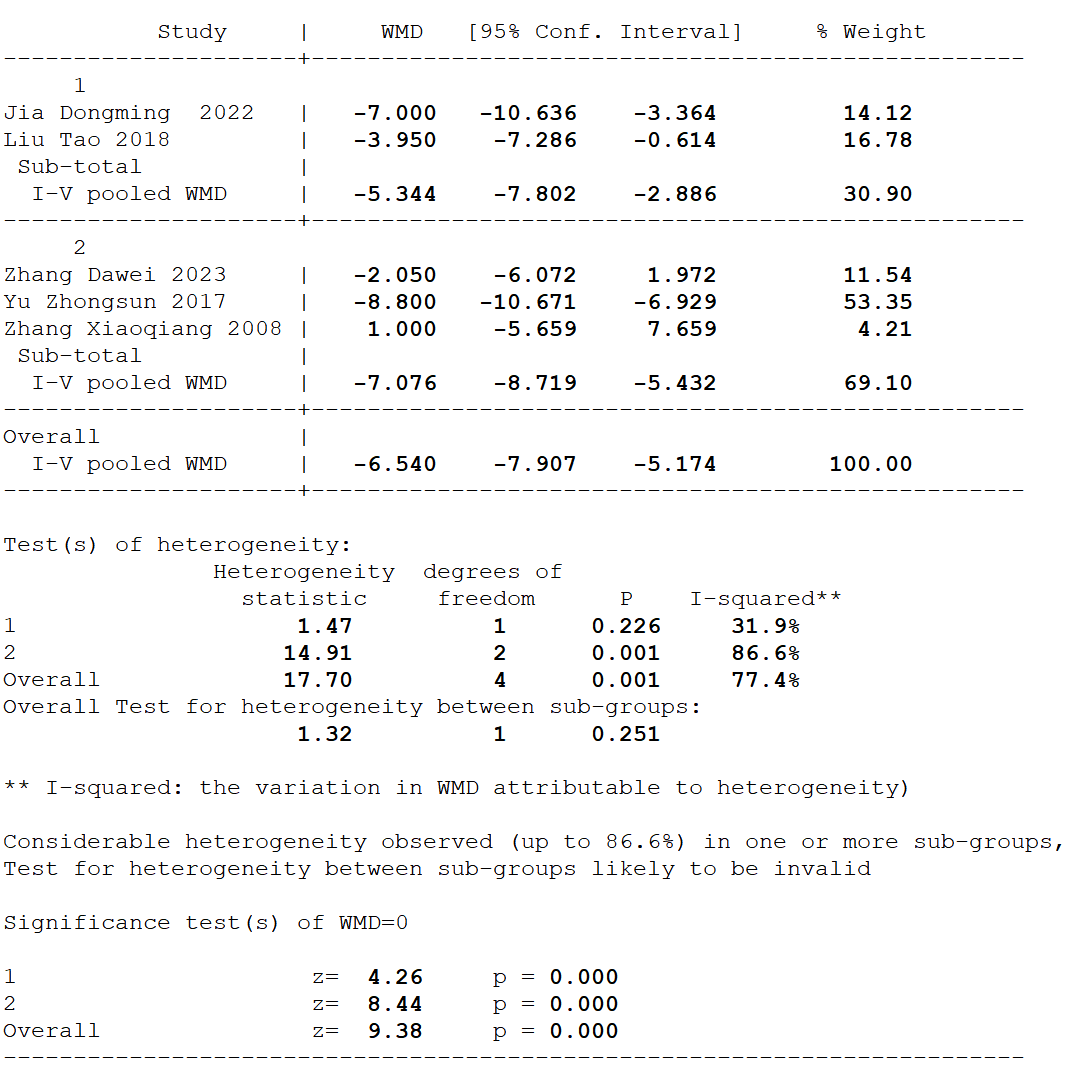

Supplement: Supplementary file 4 [file Image_4.jpeg]

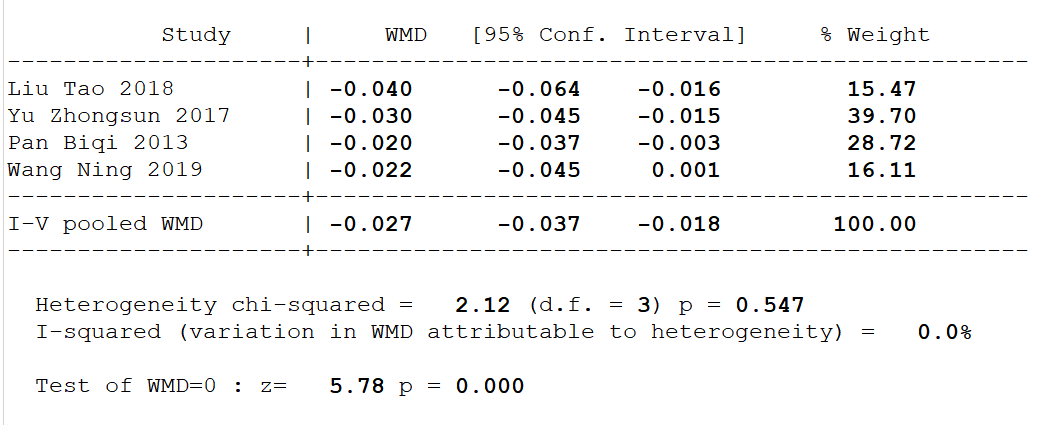

Supplement: Supplementary file 5 [file Image_5.jpeg]

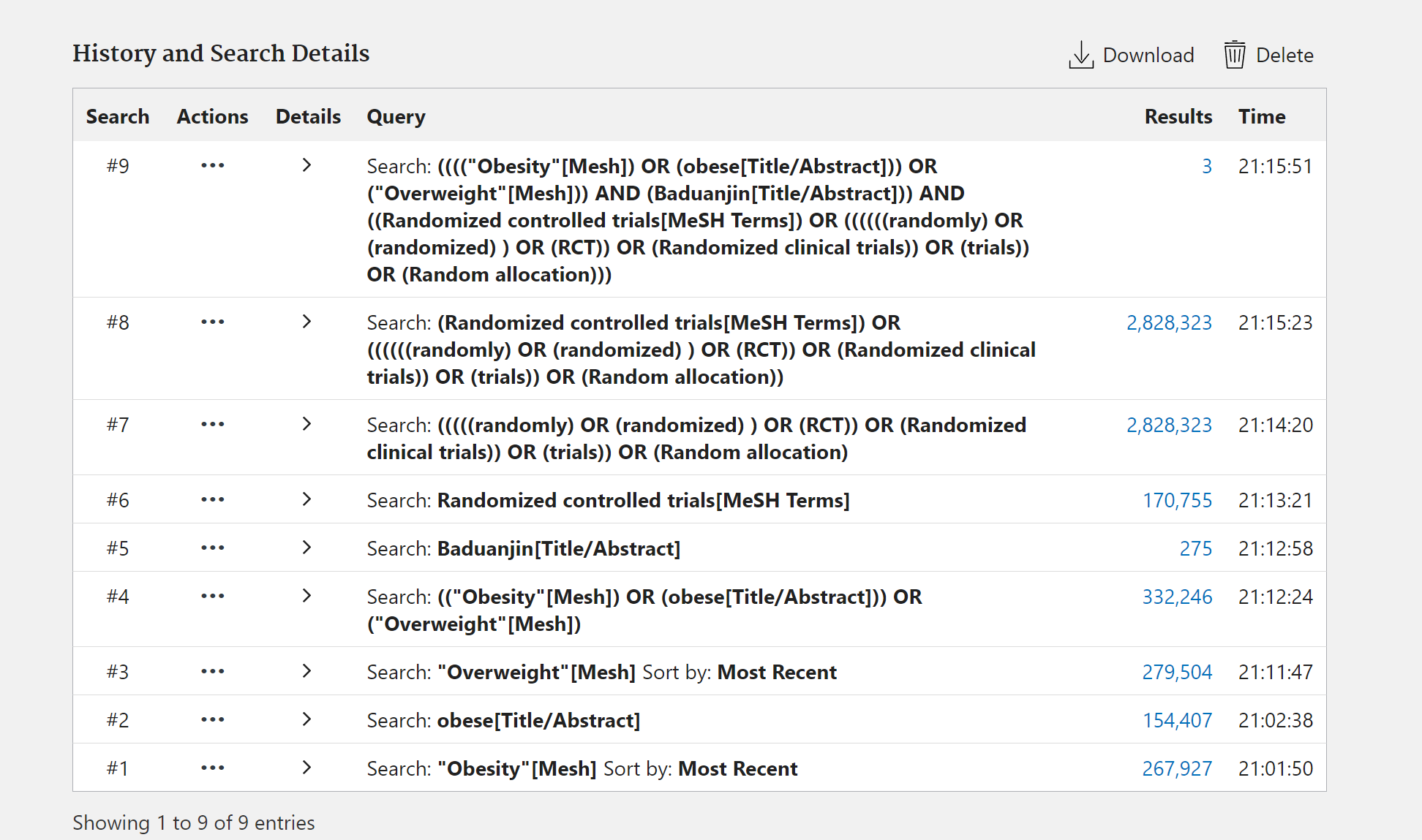

Supplement: Supplementary file 6 [file Image_6.jpeg]

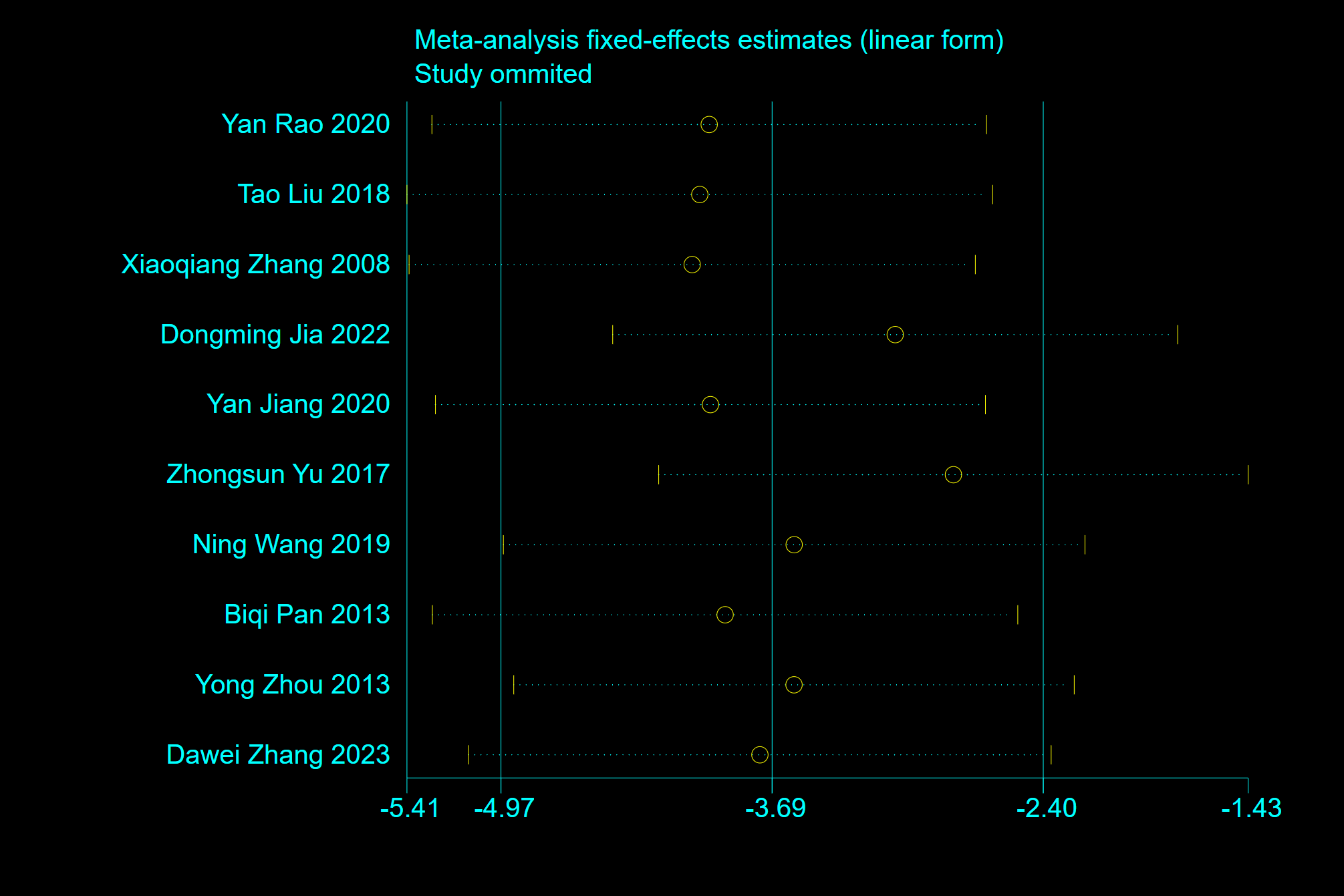

Supplement: Supplementary file 7 [file Image_7.png]
