# Supplementary material for: Efficacy of Baduanjin for obesity and overweight: a systematic review and meta-analysis
Source: Front Endocrinol (Lausanne). 2024 Jun 11;15:1338094. doi: 10.3389/fendo.2024.1338094 (PMC11196404; doi:10.3389/fendo.2024.1338094)
Supplement: Supplementary file 10 [file Table_3.docx]

| ertainty assessment | | | | | | | № of patients | | Effect | Certainty | Importance |
| --- | --- | --- | --- | --- | --- | --- | --- | --- | --- | --- | --- |
| № of studies | Study design | Risk of bias | Inconsistency | Indirectness | Imprecision | Other considerations | Experimental group | Control group | Absolute  (95% CI) |  |  |
| BW | randomised trials | serious^a^ | serious^b^ | not serious | not serious | none | 212 | 208 | MD 3.69 lower  (4.97 lower to 2.4 lower) | ⨁⨁◯◯  Low | Important |
| BMI | randomised trials | serious^a^ | serious^b^ | not serious | not serious | none | 183 | 182 | MD 1.36 lower  (1.76 lower to 0.96 lower) | ⨁⨁◯◯  Low | Important |
| WC | randomised trials | serious^a^ | serious^b^ | not serious | not serious | none | 174 | 171 | MD 5.42 lower  (6.56 lower to 4.28 lower) | ⨁⨁◯◯  Low | Important |
| HC | randomised trials | serious^a^ | serious^b^ | not serious | not serious | none | 161 | 161 | MD 3.4 lower  (4.43 lower to 2.37 lower) | ⨁⨁◯◯  Low | Important |
| WHR | randomised trials | serious^a^ | not serious | not serious | serious^c^ | none | 109 | 109 | MD 0.03 lower  (0.04 lower to 0.02 lower) | ⨁⨁◯◯  Low | Important |
